# Recipient C7 rs9292795 genotype and the risk of hepatocellular carcinoma recurrence after orthotopic liver transplantation in a Han Chinese population

**DOI:** 10.1186/s12885-021-08269-7

**Published:** 2021-05-08

**Authors:** Zhongyi Jiang, Qianwei Jiang, Xu Fang, Pusen Wang, Weitao Que, Hao Li, Yang Yu, Xueni Liu, Chunguang Wang, Lin Zhong

**Affiliations:** 1 Department of General Surgery, Shanghai General Hospital, Shanghai Jiao Tong University School of Medicine, 100 Haining Road, Shanghai, 200080 China; 2 Emergency & Critical Care Department, Shanghai General Hospital, Shanghai Jiao Tong University School of Medicine, 650 New Songjiang Road, Songjiang District, Shanghai, China

**Keywords:** C7 polymorphism, Hepatocellular carcinoma recurrence, Orthotopic liver transplantation, Prognosis

## Abstract

**Background:**

Complement component(C7) gene has been shown to influence the prognosis in Hepatocellular carcinoma (HCC) patients. The association between C7 and HCC recurrence after orthotopic liver transplantation (OLT), however, is still unknown. The purpose of this study was to evaluate whether the donor and recipient C7 gene polymorphisms are related to HCC recurrence after OLT in the Han Chinese population.

**Methods:**

A total of 73 consecutive patients with HCC who had undergone OLT, both donors and recipients, were involved in this research. A single nucleotide polymorphism of C7, rs9292795, was genotyped using Sequenom MassARRAY in the cohort. The expression of C7 and the association between C7 gene polymorphisms and HCC recurrence following OLT were analyzed by bioinformatics and statistical analysis, respectively.

**Results:**

As shown in database, the expression of C7 was higher in HCC tissues than that in normal tissues, and represented a worse prognosis. We also found that recipient C7 rs9292795 polymorphism, rather than the donor, was significantly associated with HCC recurrence after OLT. Multivariate logistic regression analysis confirmed that TNM stage (*P* = 0.001), Milan criteria (*P* = 0.000) and recipient rs9292795 genotype (TT vs AA/AT, *P* = 0.008) were independent risk factors for HCC recurrence. Furthermore, the recipient carrying AA/AT showed higher recurrence-free survival (RFS) and overall survival (OS) than that carrying TT (*P* < 0.05). In Cox proportional hazards model, TNM stage, recipient rs9292795 genotype, and Milan criteria were identified as independent factors for RFS and OS (*P* < 0.05) as well as pre-OLT serum alpha fetoprotein (AFP) level was associated with OS (P < 0.05).

**Conclusions:**

Recipient C7 rs9292795 gene polymorphism is related to the recurrence of HCC after OLT, which may be a helpful prognostic marker for HCC patients who receive OLT.

## Introduction

Hepatocellular carcinoma (HCC), the most frequent primary liver malignancy (85–90%), is the fifth most commonly diagnosed malignancy and the third leading cause of cancer-related deaths in the world over the past two decades [1]. It is estimated that there are approximately 841,000 HCC cases and 782,000 HCC-related deaths throughout the world in 2018 [1]. Most of the new cases of HCC occur in developing countries, and 55% of new cases worldwide occurred in China, which is related to the epidemic of hepatitis B virus (HBV) [2, 3]. Orthotopic liver transplantation (OLT) is considered as the most effective treatment and a routine practice for patients with HCC [4]. Owing to improved surgical techniques and new immunosuppressive drugs, OLT offers optimal outcomes by removing and replacing the entire tumor-bearing liver, and the recipient long-term survival has increased steadily, with a current 5-year survival exceeding 75% [5]. Most transplant centers have adopted the Milan, University of California San Francisco guidelines to select the best criteria for transplantation and improve the survival time after OLT [6–8] as their official tool. However, the efficacy of patients with HCC following OLT depends on the risk of the recurrence and it remains a significant challenge. In the first 3 year after OLT, the recurrence rate of patients with HCC was 8–15% [9].

Although the recent research reports illuminate signaling regulators related to early HCC recurrence [10–12], the underlyingly overall mechanism of HCC recurrence after OLT remains largely unknown. The tumor size, number, histological grade of differentiation, microvascular and macrovascular invasion, alpha-fetoprotein level, and ischemia time are proved as the predictors of HCC recurrence after OLT [8, 13–15]. However, it is more critical to explore the genetic mechanism of HCC recurrence after OLT. A previous study demonstrated that the single-nucleotide polymorphisms (SNPs) of telomere maintenance gene play a potential role in the survival of HCC patients [16]. A great many of SNPs are functional and may resulting in recurrence of HCC by altering gene expression or protein function [17, 18].

The Complement component 7(C7) gene, located on chromosome 5p13.1, is a terminal component of the complement cascade. C7 encodes a serum glycoprotein together with complement components C5b, C6, C8, and C9, which form a membrane attack complex (MAC) as part of the terminal complement pathway of the innate immune system [19, 20]. The complement system serves as the innate immune system, which is important in immune surveillance and homeostasis [21]. It has been previously reported that C7 deficiency leads to increased susceptibility to a number of human diseases [19, 20, 22, 23]. Besides, recent studies also confirmed that complement system associated with the development of carcinomas [24, 25].

The OLT patients have both donor and recipient two genetic profiles, which both may be underlying causes of HCC recurrence following OLT. A previous study demonstrated the association of C7 polymorphisms with infection after OLT [26]. We therefore aim to verify whether donor or recipient C7 polymorphisms are associated with the risk of HCC recurrence after OLT, which might be an effectively prognostic marker of HCC patient survival after OLT in a Han Chinese population.

## Materials and methods

### Patients

A total of 224 adult patients, who had undergone orthotopic liver transplantation for malignant liver disease and other reasons of hepatic failure between June 2006 and December 2013 at the Shanghai General Hospital, Shanghai Jiao Tong University, School of Medicine, were enrolled. The patients with benign liver function failure (alcohol and autoimmune hepatitis) were excluded. The inclusion criteria of these transplantation patients were as follows: aging 18 or older, compatible blood and tissue type with donors and no multiorgan combined transplantation. All the patients were Han Chinese. Thus, only 73 patients with HCC were included in the study. All patients were treated using immunosuppression regimen after OLT, which comprised cyclosporine or tacrolimus (FK506), mycophenolate, and prednisone. Patients positive for HBV-DNA were treated with Entecavir before OLT and hepatitis B immune globulin 800 IU fixed dosing during and after the operation. After leaving the hospital, all patients were monitored for tumor recurrence or metastasis using the AFP test, ultrasonography, and emission computed tomography every 3 months.

### Ethics statement

The consent for participating in this study was received from each donor and recipient. Every organ donation or transplantation was approved by the Institutional Review Board Liver Transplantation Surgery of the Shanghai General Hospital, Shanghai Jiao Tong University, School of Medicine, China, and carried out strictly in accordance with the guidelines of the Ethics Committee of the hospital, current law of the Chinese Government, and the Declaration of Helsinki [27]. No organs/tissues were procured from prisoners.

### DNA extraction and genotyping

Genomic DNA was obtained from the donor and recipient liver specimens using an AllPrep DNA/RNA Mini kit (Qiagen, Venlos, the Netherlands) following the manufacturer’s protocol. All the tissues were previously stored at − 80 °C before use. Single-nucleotide polymorphism (SNP) genotyping was performed using a Sequenom MassARRAY SNP genotyping platform (Sequenom, CA, USA).

### Gene expression analyses

The expression of C7 in liver hepatocellular carcinoma (LIHC) tissue and normal tissue as well as the effect of C7 expression level on LIHC patient survival were analyzed using UALCAN database [28].

### Statistical analysis

The OLT recipients were divided into two groups: HCC recurrence cases and controls of non-recurrence. Data were analyzed using SPSS ver.19.0 statistical software (SPSS Inc., IL, USA). The Wilcoxon signed-rank test was used to evaluate the descriptive variables which expressed as the median (range) as well as using the Pearson’s *χ*2 test or Fisher’s exact to compare the Categorical variables. The allele and genotype frequencies of C7 polymorphisms, both the donor and recipient, were calculated through SHEsis Online Version (http://analysis.bio-x.cn/myAnalysis.php) and Hardy–Weinberg equilibrium test [29]. Odds ratio and 95% confidence interval (CI) were tested using the logistic regression analysis. Kaplan–Meier survival curve analysis was performed to estimate the Recurrence-free survival (RFS) and overall survival (OS) and then adjusted by log-rank test. The independent factors associating with RFS and OS were further investigated by the multivariate Cox’s proportional hazards model following the univariate analysis. The *P* value < 0.05 was considered statistically significant.

## Results

### The basic and clinical characteristics of OLT patients

This study consisted of 73 HCC patients who had undergone OLT including 64 males and 9 females, with a median age of 49 years (aged 21–67 years). All of the patients were divided into two groups, recurrence (*n* = 29) and non-recurrence (*n* = 44). The follow-up time ranged from 2.7 to 72 months. The mean follow-up period was 30.6 months. Moreover, 57 patients had the etiology of liver cirrhosis. As we found that the Milan criteria, TNM stage, tumor size, and microvascular invasion were statistically significant (*P* < 0.05). The patient demographic, and clinical and pathological characteristics are listed in Table 1.

**Table 1:** Demographic, clinical and pathological characteristics of the two groups of the HCC patients

Parameter	Recurrence group (*n* = 29)	Non-recurrence group (*n* = 44)	*P* value
Recipient age	48(21–67)	49.5(33–62)	0.811
≥ 50	13(44.8%)	22(50.0%)	
<50	16(55.2%)	22(50.0%)	
Recipient gender			1.000
Male	25(86.2%)	39(88.6%)	
female	4(13.8%)	5(11.4%)	
Hepatitis B			0.207
Yes	25(86.2)%)	42(95.5%)	
No	4(13.8%)	2(4.5%)	
Cirrhosis			0.154
Yes	20(69.0%)	37(84.1%)	
No	9(31.0%)	7(15.9%)	
Milan criteria			0.000
In	2(6.9%)	29(65.9%)	
Out	27(93.1%)	15(34.1%)	
TNM stage			0.000
1–2	12(41.4%)	38(86.4%)	
3–4	17(58.6%)	6(13.6%)	
Tumor size (cm)			0.000
> 5	26(89.7%)	18(40.9%)	
≤ 5	3(10.3%)	26(59.1%)	
Multinodular type			0.219
YES	15(51.7%)	30(68.2%)	
NO	14(48.3%)	14(31.8%)	
Pre-OLT serum AFP level			0.165
≤ 400 (ng/ml)	19(65.5%)	36(81.8%)	
> 400 (ng/ml)	10(34.5%)	8(18.2%)	
Microvascular invasion			0.000
Yes	14(48.3%)	2(4.5%)	
No	15(51.7%)	42(95.5%)	
Macrovascular invasion			0.425
YES	4(57.1%)	3(42.9%)	
NO	25(37.9%)	41(62.1%)	

*Abbreviations*

*HCC* hepatocellular carcinoma, *TNM* tumor node metastasis, *AFP* alpha-fetoprotein, *OLT* orthotopic liver transplantation

### C7 rs9292795 genotype distribution and its association with the HCC recurrence after OLT

As shown in Table 2, the distribution of C7 genotypes is presented. We tested whether all genotype frequencies satisfy the Hardy–Weinberg equilibrium. There was no statistically significant in the frequencies of distribution of C7 genotype polymorphism of donor between recurrence group and non-recurrence group. On the contrary, the difference is quite significant in the recipient group. In addition, we also observed that the dominant model was the most significant, and the recurrence rate was notably higher in patients with the recipient rs9292795 TT genotype than in those with AA/AT genotype (69% vs 31%, *P* = 0.009).

**Table 2:** Recipient and donor C7 genotype distribution and the association with HCC recurrence

	Genotype distribution, n (%)		*P* value	HWE *P* value
	Recurrence (*n* = 29)	Non-recurrence (*n* = 44)		
Recipient rs9292795				
AA	1(3.4%)	8(18.2%)		
AT	8(27.6%)	20(45.5%)	0.016	0.858
TT	20(69.0%)	16(36.4%)		
Recessive model				
AA	1(3.4%)	8(18.2)		
AT/TT	28(96.6%)	36(81.8%)	0.078	
Dominant model				
TT	20(69.0%)	16(36.4%)	0.009	
AA/AT	9(31.0%)	28(63.6%)		
Additive model				
AT	8(27.6%)	20(45.5%)		
AA/TT	21	24	0.146	
Donor rs9292795				
AA	3 (10.3%)	4(9.1%)		
AT	16(55.2%)	23(52.3%)		
TT	10(34.5%)	17(38.6%)	0.933	0.355
Recessive model				
AA	3(10.3%)	4(9.1%)		
AT/TT	26(89.7%)	40(90.9%)	1.000	
Dominant model				
TT	10(34.5%)	17(38.6%)		
AA/AT	19(65.5%)	27(61.4%)	0.087	
Additive model				
AT	16(55.2%)	23(52.3%)		
AA/TT	13(44.8%)	21(47.7%)	1.000	

*Abbreviations: HCC* hepatocellular carcinoma, *HWE* Hardy–Weinberg equilibrium

### Risk factors for recurrence: multivariate logistic regression analysis

The potential risk factors for HCC recurrence after OLT, testing by the Pearson’s *χ*2 test or Fisher’s exact test (*P* < 0.05), are presented in Table 1. Clinical parameters such as Milan criteria, TNM stage, tumor size, multinodular type, microvascular invasion, and macrovascular invasion were involved. On account of TNM staging including the size of tumor, the variable tumor size was excluded to avoid collinearity. Finally, genetic factors (TT vs. AA/AT) were selected in the model in which the results could be more credible. Collinearity diagnostics testing had shown no collinearity (tolerance > 0.1) of all these variables. According to their clinical significance, we defined appropriate cutoff levels. Multivariate logistic analysis indicated that HCC recurrence after OLT significantly associated with the following factors: TNM stage [OR = 8.302 (1.723–40.008), *P* = 0.001], Milan criteria [OR = 26.100 (5.453–124.921), *P* = 0.000], and recipient rs9292795 [TT vs AA/AT, OR = 3.889 (1.433–10.551), *P* = 0.008] (Table 3).

**Table 3:** Multivariate logistic regression analysis of risk factors associated with HCC recurrence

Factors	Odds ratio (95%CI)	*P* value
TNM stage (0 = stage 1–2, 1 = stage 3–4)	8.302 [1.723–40.008]	0.001
Milan criteria(1 = out, 0 = in)	26.100 [5.453–124.921]	0.000
Recipient rs9292795(1 = AA/AT,2 = TT)	3.889 [1.433–10.551]	0.008

*Abbreviation: CI* confidence interval

### Database analyses, Univariate cox hazard regression analysis and Kaplan–Meier survival curves to detect risk factors for recurrence

**Fig. 1:**
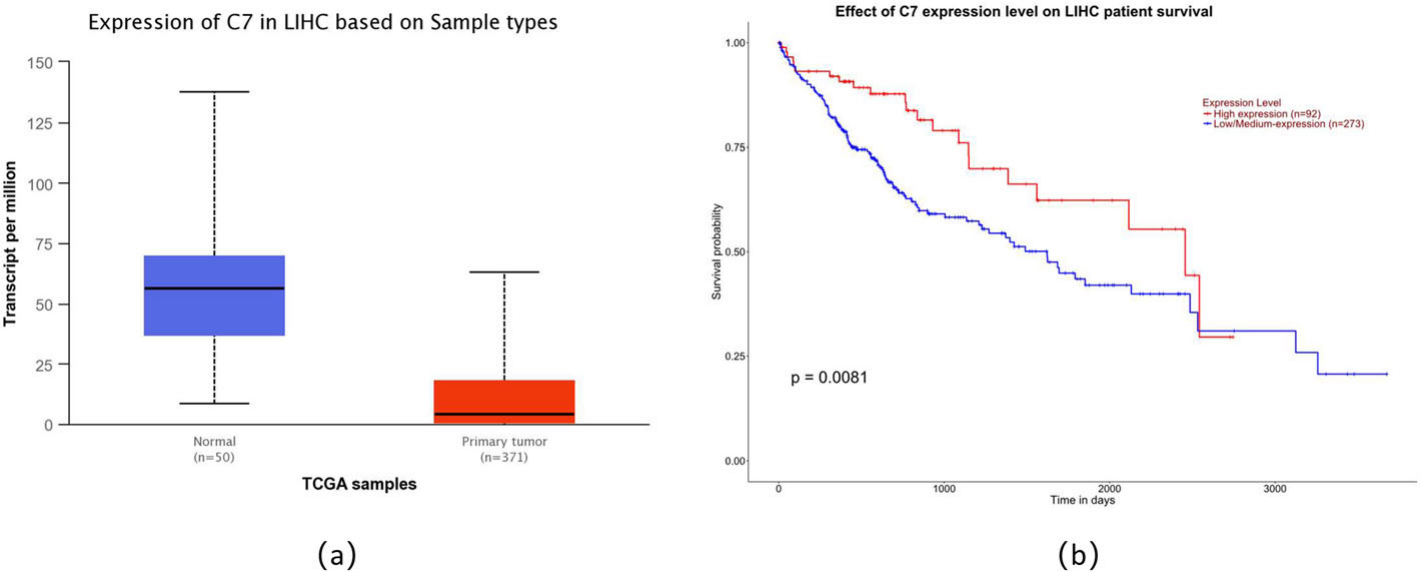
Expression of C7 in human tissue database. **a** Expression of C7 gene is lower in LIHC primary tumor(*n* = 371) than in normal tissue(*n* = 50). **b** Kaplan-Meier analysis of overall survival (OS) grouped by high C7 and low C7 levels

**Fig. 2:**
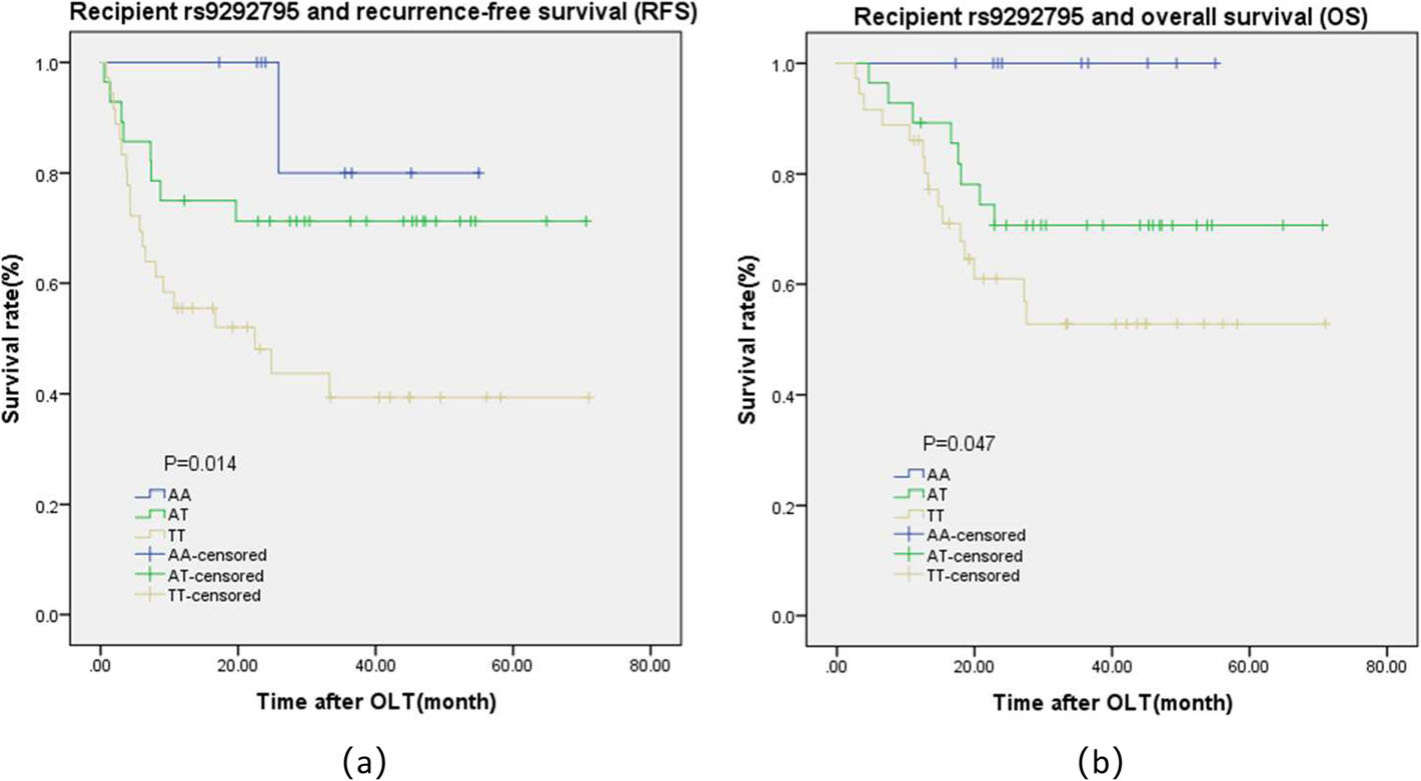
Kaplan–Meier survival estimates of **a** recurrence-free survival (RFS) and **b** overall survival (OS) between the different recipient genotypes (AA, AT, and TT) of the recipient

**Fig. 3:**
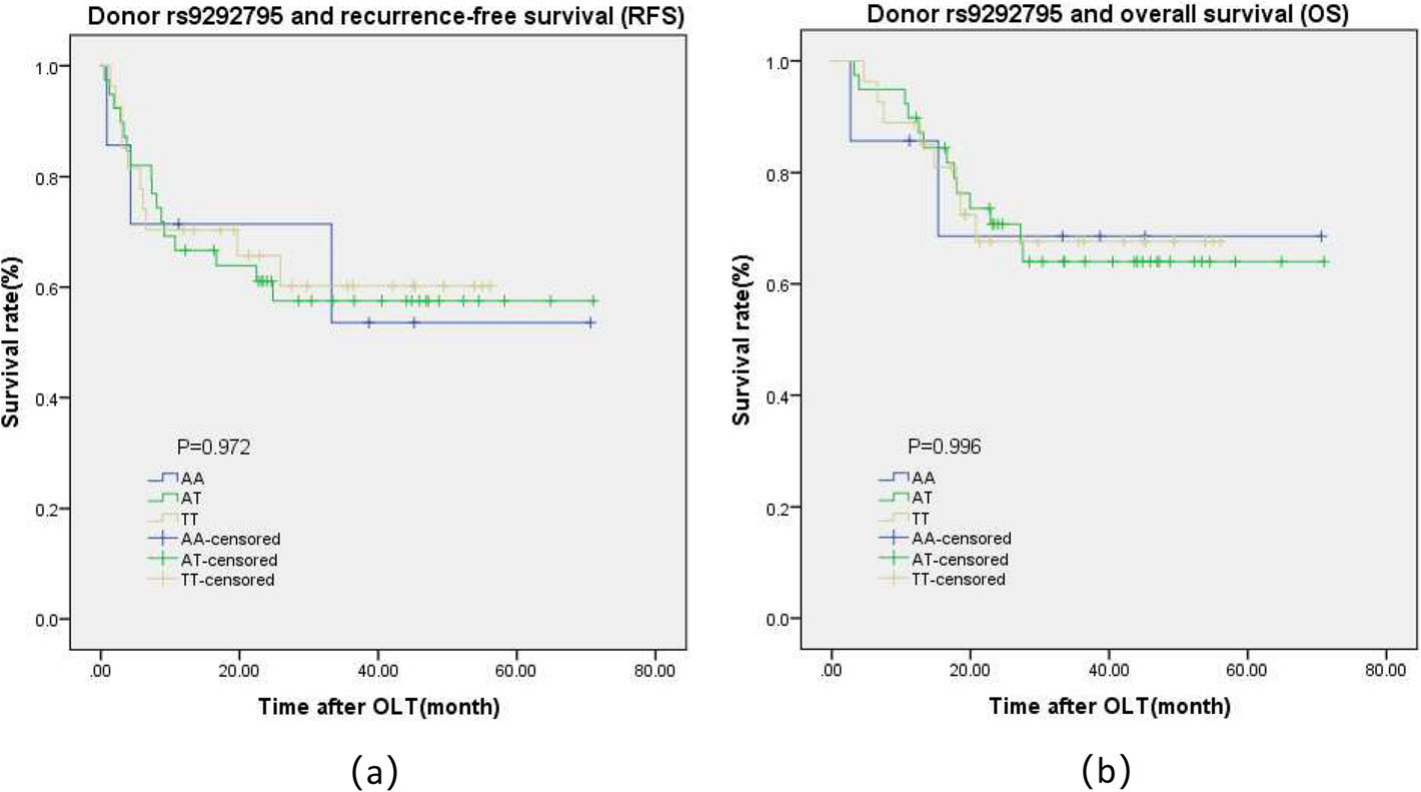
Kaplan–Meier survival estimates of **a** recurrence-free survival (RFS) and **b** overall survival (OS) between the different recipient genotype (AA, AT, and TT) of the donor

We further comprehensive analysis TCGA datasets using the UALCAN database objective to validate our findings. As shown in Fig.1a, detection of 371 LIHC tissues and 50 normal tissues demonstrated that C7 was lower expression in LIHC tissues. We also found that LIHC patients with low/medium-expression of C7 had a worse prognosis (Fig.1b). The detailed findings of the univariate Cox regression analysis for each risk factor are listed in Table 4. It is worth nothing that Milan criteria, TNM stage, tumor size, microvascular invasion, pre-OLT serum AFP level, and recipient rs9292795 (TT vs. AA/AT) were considered to be statistically significant factors for both RFS and OS (*P* < 0.05). Besides, RFS and OS were analyzed by the Kaplan–Meier survival estimates and the log-rank test considering the genetic factors. Significant differences were found in recipient C7 RFS and OS among recipients carrying the AA, AT, and TT alleles (*P* = 0.014 and *P* = 0.047, respectively; Fig.2a, b). Shown here, we observed that the RFS and OS in the recipient AA/AT group were obviously highly than in the TT group. In contrast, no significant differences were observed when the C7 SNP polymorphisms of the donor were analyzed and their association with the survival after transplantation was assessed (no significant differences in RFS and OS between the donor C7 genotypes; *P* = 0.972 and *P* = 0.996, respectively; Fig. 3a, b).

**Table 4:** Prognostic factors associated with RFS and OS in the univariate Cox analysis

Parameter	RFS, *P* -value	OS, *P* -value
Recipient age(<50/≥50)	0.531	0.284
Gender (Male/ Female)	0.831	0.891
Hepatitis B (yes/no)	0.108	0.953
Cirrhosis (yes/no)	0.095	0.306
Milan criteria (in/out)	0.000*	0.000*
TNM stage(1–2/3–4)	0.000*	0.000*
Tumor size (cm) (≤5/> 5)	0.000*	0.001*
Multinodular type (yes/no)	0.111	0.172
Microvascular invasion (yes/no)	0.000*	0.000
Macrovascular invasion (yes/no)	0.367	0.627
serum AFP level(≤400/> 400)	0.060	0.003*
Recipient rs9292795(TT vs AA/AT)	0.005*	0.037*
Recipient rs9292795(TT vs AA vs AT)	0.014*	0.047*
Donor rs9292795(TT vs AA/AT)	0.825	0.927
Donor rs9292795(TT vs AA vs AT)	0.972	0.996

*Abbreviations:*

*HCC* hepatocellular carcinoma, *TNM* tumor node metastasis, *AFP* alpha-fetoprotein, *RFS* recurrence-free survival, *OS* overall survival, *P < 0.05

### Multivariate cox regression models

We further investigated the prognostic factors associating with RFS and OS by the multivariate Cox model. The factors with corresponding *P* < 0.05 in the univariate analysis were enrolled. Collinearity diagnostics testing performed as we mentioned above. As shown in Table 5, TNM stage (*P* < 0.001), recipient rs9292795 (*P* = 0.003), and Milan criteria (*P* = 0.000) were independent factors for RFS. Furthermore, AFP (*P* = 0.001), TNM stage (*P* < 0.001), recipient rs9292795 (*P* = 0.001), and Milan criteria (*P* = 0.001) were independent factors for OS among patients with HCC who had undergone OLT. Recipient rs9292795 TT type results in shorter OS, RFS, and higher risk for recurrence.

**Table 5:** Prognostic factors associated with RFS and OS in the multivariate Cox regression analysis

	Hazard ratio (95% CI)	*P* value
RFS		
Milan criteria	16.408 [3.869–69.592]	0.000
Recipient rs9292795(1 = TT,2 = AA/AT)	3.672[1.574–8.566]	0.003
TNM stage(1 = 1-2stage,2 = 3-4stage)	5.738[2.305–14.288]	0.000
OS		
AFP	3.085[1.012–9.408]	0.048
Milan criteria	11.078[2.587–47.443]	0.001
Recipient rs9292795(1 = TT,2 = AA/AT)	4.415[1.792–10.879]	0.001
TNM stage(1 = 1-2stage,2 = 3-4stage)	10.318[3.016–35.294]	0.000

*Abbreviations:*

*CI* confidence interval, *TNM* tumor node metastasis, *AFP* alpha-fetoprotein

## Discussion

The Complement component 7(C7) located on chromosome fragment 5p13.1–13.3, which might involve in the progression of cancer if the copy number variated [20]. In this study, we investigated the expression of C7 in HCC tissue as well as the association between *C7* gene variations and HCC recurrence following OLT. Based on the UALCAN database validated that *C7* gene was lower expressed in HCC tissues with shorter OS in patients. Obviously, our findings indicated that the recipient polymorphism of C7 rs9292795 had significant association with HCC recurrence after OLT. RFS and OS in the recipients C7 rs9292795 TT type group were remarkably shorter than in the AA/AT group, especially higher HCC recurrence after OLT. The results suggested that recipient C7 rs9292795 might help establish a potentially effective system for prognosis and organ allocation.

The serum glycoprotein that C7 encodes is one of the central components of membrane attack complex (MAC), which is part of the terminal complement pathway of the innate immune system [20]. A previous study indicates that MAC as the product of complement activation may promote tumor growth [24]. In addition, MAC activating complement pathway plays an important role in the pathogenesis of a variety of liver diseases, such as defend foreign pathogens by lysing them directly [30]. Furthermore, Zhong et al. [26] had demonstrated that the association of C7 polymorphisms with infection after OLT in a Han Chinese population. Therefore, we hypothesis that the C7 polymorphisms may associated with the HCC recurrence following OLT.

According to previous reports, the expression of C7 was remarkably reduced in oesophageal carcinoma [19] and non-small cell lung cancer (NSCLC) [20] tissues. Moreover, multivariate cox regression analysis showed low-expressed C7 had a higher risk for recurrence in NSCLC patients [20]. Our results demonstrated that the recipients with the C7 rs9292795 TT genotype had worse survival and higher incidence of HCC recurrence following OLT compared with those carrying the C7 rs9292795 AA/AT genotype. Combined with existing reports, our study suggested that C7 rs9292795 is a functional SNP and may contribute to the HCC recurrence after OLT. However, the mechanisms of how the C7 rs9292795 TT genotype is involved in HCC recurrence following OLT is complicated and need more in-depth research to reveal them.

Similar to the previous study, we found TNM stage and Milan criteria were independent factors for HCC recurrence after OLT in clinical analysis [14]. Besides, Milan criteria, TNM stage, microvascular invasion, pre-OLT serum AFP level were independent factors for patient prognosis.

Notably, the present study had some limitations. First, the sample size was relatively small, and the follow-up period was not long enough. Second, most patients had HBV-related liver diseases. Hence, further studies should involve large samples with various HCC causes (hepatitis C virus, aflatoxin, and alcohol). Third, the relationship between the C7 gene polymorphism, C7 gene expression, and HCC recurrence after OLT is complicated and hence difficult to uncover. Future studies should focus on gene and protein functions to fully understand C7and the recurrence of liver carcinoma.

In summary, our findings demonstrated that C7 gene polymorphism obviously associated with HCC recurrence in patients who had undergone OLT. C7 rs9292795 TT genotype of the recipient rather than of the donor is of greater value in predicting the risk of HCC recurrence after OLT. Furthermore, C7 rs9292795 may represented an independent factor for long-term prognosis of patients with HCC following OLT.

## Data Availability

Data sharing is not applicable to this article as no datasets were generated during the current study.

## Abbreviations

AFP: serum alpha fetoprotein
C7: Complement component
CI: confidence interval
HBV: hepatitis B virus
HCC: Hepatocellular carcinoma
HWE: Hardy–Weinberg equilibrium
LIHC: liver hepatocellular carcinoma
MAC: membrane attack complex
OLT: orthotopic liver transplantation
OR: odds ratio
OS: overall survival
RFS: recurrence-free survival
SNPs: single nucleotide polymorphisms
TNM: tumor node metastasis
